# Public Charities

**Published:** 1856-04

**Authors:** 


### PUBLIC CHARITIES.

It is well known to the profession, if it is not so familiar to community at large, that the medical man dispenses more charities than any other member of society. Not a day passes but that he is required to bestow dollars to the indigent and suffering poor. This bestowment is made too, generally under the most unfavorable and disagreeable circumstances, to which he would much prefer to put his hand into his pocket and pay the nominal equivalent of his labor in money. But he has not this choice, for humanity demands still greater sacrifices. He must leave his bed in the night, wander off in the cold, in a storm perhaps, to some wretched hovel and there remain without the common comforts of life, in communion and contact with some poor miserable unfortunate, whose destitution often thwarts his best directed efforts for recovery or relief. This exposure results, in many instances, in a severe affliction to himself, from which he is confined to his house for days, and so soon as he is able to be abroad, some benevolent committee sues him for contributions to some charitable object, to which his liberality and charity inclines him to contribute of his hard earnings. He is appealed to in some other case of destitution during the day, and in addition to this he finds some one of his indigent patients destitute of proper nourishment, and his hand is in his pocket for means to supply the needed materials. Thus it is from morning until night, from night until morning, day after day, week after week, and from year to year,—his services and his purse are ever contributing to charitable objects.

Notwithstanding this continued drain upon and this never-ending demand for aid, when some public enterprise is set on foot in which his professional services are necessary, he is screwed down, and jewed down, by public functionaries to a pitiful sum for his services, and it is even then doled out to him with a niggardly liberality, even when, according to agreement, his services are not half requited. All this, too, to make an exhibit of care and economy in expenditures. What an outrage it is to begin a system of mean parsimony in matters of charity. How high souled such philanthropy! how magnanimous such conduct! When niggardly parsimony and close and narrow calculations enter into the management of benevolent enterprises, they become degraded from their design, and are caricatures of that spirit of christian charity which dictates their existence. In this particular, those entrusted with their management, exceed the wishes of the masses of the people. They may, and should, require economy in ordinary public expenditures, but in all matters of benevolence, and in the management of humane enterprises, we have never yet heard a single complaint, and our experience for many years, where the most liberal bestowments were made for the comfort of the indigent afflicted, fully justifies this assertion.

The medical attention upon public hospitals, alms-houses, and poor houses should be properly remunerated, without allowing competition to lower the price of services.

The practice of giving the medical charge of these to the lowest bidder in indeed inhuman. The poor are entitled to the best skill, and yet, in this way, it is seldom secured. There are those often, whose limited acquirements, do not receive encouragement in private practice, and are driven, by necessity, to take charge of these public charities as a means of livelihood. But is this just? Is it in accordance with the spirit of humanity or philanthropy? Most assuredly not, and those whose taxes sustain these enterprises, would not endorse such a trickstering system, under the plea of economy. Such practices are degrading to the profession, a crying injustice to the unfortunate poor, and stains upon the spirit of humanity and charity.

We know that there are public functionaries who do not act illiberally nor unjustly, as we could point to some to whom these remarks are by no means applicable, but in times past there have been cases in which there has been exhibited a parsimonious and ungenerous spirit in making allowances for such services.

